# The Risk of Fractures Associated with Thiazolidinediones: A Self-controlled Case-Series Study

**DOI:** 10.1371/journal.pmed.1000154

**Published:** 2009-09-29

**Authors:** Ian J. Douglas, Stephen J. Evans, Stuart Pocock, Liam Smeeth

**Affiliations:** 1 Non Communicable Disease Unit, Department of Epidemiology & Population Health, London School of Hygiene & Tropical Medicine, London, United Kingdom; 2 Medical Statistics Unit, Department of Epidemiology & Population Health, London School of Hygiene & Tropical Medicine, London, United Kingdom; University of Sydney, Australia

## Abstract

**Background:**

The results of clinical trials have suggested that the thiazolidinedione antidiabetic agents rosiglitazone and pioglitazone are associated with an increased risk of fractures, but such studies had limited power. The increased risk in these trials appeared to be limited to women and mainly involved fractures of the arm, wrist, hand, or foot: risk patterns that could not be readily explained. Our objective was to further investigate the risk of fracture associated with thiazolidinedione use.

**Methods and Findings:**

The self-controlled case-series design was used to compare rates of fracture during thiazolidinedione exposed and unexposed periods and thus estimate within-person rate ratios. We used anonymised primary care data from the United Kingdom General Practice Research Database (GPRD). All patients aged 40 y or older with a recorded fracture and at least one prescription for a thiazolidinedione were included (*n* = 1,819). We found a within-person rate ratio of 1.43 (95% confidence interval [CI] 1.25–1.62) for fracture at any site comparing exposed with unexposed periods among patients prescribed any thiazolidinedione. This association was similar in men and women and in patients treated with either rosiglitazone or pioglitazone. The increased risk was also evident at a range of fracture sites, including hip, spine, arm, foot, wrist, or hand. The risk increased with increasing duration of thiazolidinedione exposure: rate ratio 2.00 (95% CI 1.48–2.70) for 4 y or more of exposure.

**Conclusion:**

Within individuals who experience a fracture, fracture risk is increased during periods of exposure to thiazolidinediones (both rosiglitazone and pioglitazone) compared with unexposed periods. The increased risk is observed in both men and women and at a range of fracture sites. The risk also increases with longer duration of use.

*Please see later in the article for the Editors' Summary*

## Abstract 2

Ian Douglas and colleagues analyze records from the UK General Practice Research Database, and find that among individuals prescribed thiazolidinediones who develop a fracture, fractures are more common during periods of thiazolidinedione exposure than unexposed periods.

## Editors' Summary

**Background:**

Worldwide, nearly 250 million people have diabetes and this number is increasing rapidly, particularly in developing countries. Diabetes is a chronic disease characterized by dangerous amounts of sugar (glucose) in the blood. Blood-sugar levels are normally controlled by insulin, a hormone that the pancreas releases when blood-sugar levels rise after eating (the digestion of food produces glucose). Blood-sugar control fails in people with diabetes because they make no insulin (type 1 diabetes) or because the fat cells and muscle cells that usually respond to insulin by removing sugar from the blood have become insulin insensitive (type 2 diabetes). Type 1 diabetes is treated with insulin injections; type 2 diabetes—the most common type of diabetes—is controlled with diet, exercise, and antidiabetic pills, drugs that help the pancreas make more insulin (for example, sulfonylureas) or that make cells more sensitive to insulin (for example, thiazolidinediones). Long-term complications of diabetes include kidney failure, blindness, and nerve damage, and an increased risk of developing cardiovascular problems, including heart disease and strokes.

**Why Was This Study Done?:**

Thiazolidinediones are widely used to treat type 2 diabetes but, worryingly, these drugs seem to increase people's risk of developing cardiovascular problems. In addition, they may increase the risk of bone fractures although the evidence for this particular association is limited. Given the large number of people with diabetes, it is important to understand the benefits and risks of thiazolidinedione treatment of diabetes as fully as possible. In this self-controlled case-series study, therefore, the researchers investigate the risk of fracture associated with the use of rosiglitazone and pioglitazone (two thiazolidinedione antidiabetic agents). A “self-controlled case-series study” compares how often an event (in this case, a fracture) occurs (the event's “rate”) in a population of individuals during a period of time when the individuals are not exposed to a medical intervention (in this case, treatment with thiazolidinediones) to its rate during a period when they are exposed to the intervention. Because each person acts as their own control, this study design helps to eliminate the possibility that unrecognized characteristics that vary between people (“confounders”) are responsible for differences in the event rate rather than the intervention itself.

**What Did the Researchers Do and Find?:**

The researchers identified 1,819 people aged 40 years or older with a recorded fracture and at least one prescription for a thiazolidinedione by searching the UK General Practice Research Database, which contains personal and health data for more than 6 million UK residents. They compared these people's fracture rate during periods when they were taking a thiazolidinedione to their fracture rate when they weren't taking one of these drugs. After adjusting for age (age is a potential confounder because the risk of fractures increases with age and all the patients were older during their exposed period than during their unexposed period), the rate ratio for fracture at any site in patients during thiazolidinedione-exposed periods compared with thiazolidinedione-unexposed periods was 1.43. That is, nearly one and half times as many fractures occurred when people were taking thiazolidinediones than when they were not taking these drugs. The association between taking thiazolidinediones and the risk of fracture was similar in men and women and at several fracture sites but increased with the length of thiazolidinedione exposure.

**What Do These Findings Mean?:**

These findings suggest that taking thiazolidinediones is associated with an increased risk of fracture at a wide range of sites in both men and women. They also suggest that the risk of fracture increases with treatment duration. These findings do not prove that thiazolidinediones cause fractures because, despite the self-controlled case-series design of this study, it remains possible that the people who have fractures share some unknown characteristic that affects their chances of breaking a bone. The accuracy of the findings is also dependent on the quality of the data in the General Practice Research Database. Nonetheless, these results are in keeping with the findings of clinical trials and other observational studies, suggesting they represent a real effect of treatment with thiazolidinediones. Although it is not clear yet how thiazolidinediones weaken bones, these findings need to be included in the ongoing debate about the risks and benefits of the treatment of type 2 diabetes with thiazolidinediones.

**Additional Information:**

Please access these Web sites via the online version of this summary at http://dx.doi.org/10.1371/journal.pmed.1000154.

- The International Diabetes Federation provides information about all aspects of diabetes
- The US National Diabetes Information ClearingHouse provides detailed information about diabetes (including information on medicines for diabetes) for patients, health-care professionals, and the general public (in English and Spanish)
- The UK National Health Service also provides information for patients and carers about type 2 diabetes (in several languages)
- MedlinePlus provides links to further resources and advice about diabetes and diabetes medicines (in English and Spanish)
- Information about the UK General Practice Research Database and about the self-controlled case-series method is available
- More information is available where the research was done at The London School of Hygiene & Tropical Medicine

## Introduction

The use of thiazolidinedione antidiabetic agents has become widespread in the treatment of type 2 diabetes since their introduction in the late 1990s, although recently the safety of this class of medicines has been called into question [1]–[4]. Although most attention has focused on the possible vascular effects of thiazolidinediones, the ADOPT trial of rosiglitazone also detected a 2-fold increased risk of fractures among patients treated with rosiglitazone [5]. Similar effects were seen in a pooled analysis of pioglitazone trials [6], suggesting a possible class effect. Surprising—and unexplained—features of the observed increased fracture risk in clinical trials were that the risk appeared to mainly involve fractures of the arm, hand, wrist, or foot, and that the increased risk was restricted to women. However, the individual clinical trials did not have adequate statistical power to reliably assess fracture risk. People prescribed and not prescribed thiazolidinediones are likely to differ in ways that are difficult to measure and control for, making most observational studies subject to confounding and difficult to interpret. We therefore applied the self-controlled case-series design (a within-person approach) to assess the risk of fracture associated with thiazolidinedione use. This approach eliminates fixed (non-time-varying) between-person confounding, which is not the case with alternative case-control or cohort designs [7]. The study was based on primary care computerized clinical records from the United Kingdom-based General Practice Research Database (GPRD).

## Methods

### The General Practice Research Database

The GPRD contains information from over 6 million patients registered at over 400 computerised general practice surgeries in the UK [8],[9]. Continuous information is recorded for each patient including a record of each consultation, any diagnoses made, all prescribed medicines, and basic demographic data. The geographical distribution and size of GP practices represented in GPRD is largely representative of the population of England and Wales, and the individuals registered on the database are representative of the whole UK population in terms of age and sex [10]. The quality of data held in the GPRD is subject to rigorous checks and regular audits and it has been successfully used to conduct over 500 peer-reviewed published studies. The information obtained from the database is entirely anonymous. Ethical approval for this study was obtained from the Independent Scientific Advisory Group of the General Practice Research Database and the London School of Hygiene and Tropical Medicine Ethics Committee.

### Selection of Participants

Patients were selected from the population registered with the GPRD up to 4 April 2007. Eligible participants were all those with both a diagnosis of fracture and a first recorded thiazolidinedione prescription at least 12 mo after initial registration with the GPRD.

### Self-controlled Case-Series Design

The self-controlled case-series design method is derived from rate modelling using a Poisson distribution and is therefore comparable with cohort methodology. Only cases are included; thus the modelling is conditional on people experiencing the outcome in question (here, diagnosis of fracture) at some stage. It relies on within-person comparisons in a population of individuals who have both the outcome and exposure of interest [7],[11]. Incidence rate ratios are derived, comparing the rate of events during exposed periods of time with the rate during all other observed time periods. A major advantage of this design is that the potential confounding effect of both recorded and unrecorded fixed characteristics that vary between individuals, such as frailty, is removed.

**Figure 1:**
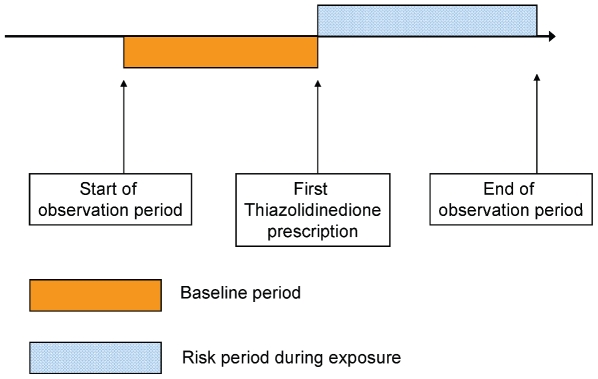
Representation of study design. The figure illustrates a single individual prescribed a thiazolidinedione during their observation period. All participants included in the analysis had at least one prescription for a thiazolidinedione and at least one fracture. The rate ratios presented are pooled estimates derived from the rate of events during the risk (exposed) periods divided by the rate of events during the baseline periods. Age is adjusted for at all stages of the analysis.

For each participant, we identified all thiazolidinedione prescriptions. The length of exposure following each individual prescription was calculated using information recorded in GPRD on pack size and dosing frequency. From all prescription records where this information was available, the median length of exposure from a single prescription was calculated. This median duration was imputed for all individual prescriptions where duration was not recorded. Thiazolidinedione treatment was assumed to be continuous where any apparent treatment break was less than 60 d, to allow for partial noncompliance and situations where patients may have built up treatment stocks. Follow-up time was then censored at the earliest of any treatment break longer than 60 d or the end of recorded follow up in the database. A mechanism for thiazolidinedione-induced fractures has not yet been established and so it is not clear whether the influence of thiazolidinediones is short lived or maintained long after treatment cessation. By censoring follow up at a prolonged treatment break we do not make any assumptions about the duration of influence and therefore avoid bias owing to misclassification of exposure. The period prior to the first thiazolidinedione prescription was classified as the baseline, unexposed period. A representation of exposure periods is shown in Figure 1.

Recorded fractures were classified according to fracture site (ankle, arm, chest/rib, face, foot, hand, hip, leg, pelvis, shoulder, skull, wrist, spine, multiple sites, unknown site). Where more than one fracture was recorded for a patient, we assumed each fracture was incident if it occurred at a different site to the previous fracture, or was recorded more than 6 mo after a fracture at the same site.

Analysis using the self-controlled case-series design inherently takes duration of follow up into account. We estimated relative rate ratios for fracture using conditional Poisson regression with Stata Version 9 software (StataCorp), adjusting for age at first thiazolidinedione prescription in 1-y bands. We first assessed the impact of exposure to any thiazolidinedione on fracture at any site. We also looked for evidence of sex differences, the differential effects of rosiglitazone and pioglitazone, the effect of thiazolidinedione exposure duration, and the effect on fractures at specific sites: (1) arm, wrist, hand, and foot; (2) hip; (3) spine.

For validation purposes we also assessed the association between treatment with a sulphonylurea and fracture at any site as we had no a priori reason to suspect an association. The same study population was used, but exposed periods were defined as periods where the patient was receiving any sulphonylurea. For this analysis, follow up was censored at the date of first thiazolidinedione prescription.

## Results

We identified 1,819 patients in the GPRD prescribed at least one thiazolidinedione and with a record of at least one fracture and background details for these patients are shown in Table 1. 1,356 patients received only prescriptions for rosiglitazone, 389 received only pioglitazone, and the remaining 74 received prescriptions for both rosiglitazone and pioglitazone at different times. 990 (54%) were female and the mean age at first thiazolidinedione exposure was 57.9 y for men and 65.4 y for women. The mean duration of unexposed follow up prior to thiazolidinedione use was 9.5 y, and the mean duration of exposure to a thiazolidinedione was 2.3 y. Arm, foot, wrist, or hand fractures were recorded for 905 patients, hip fractures for 150 patients, and spine fractures in 66 patients. The remaining 733 patients had fractures at other sites. Patients with hip and spine fractures tended to be older at first thiazolidinedione prescription: mean age of 70.5 y and 65.6 y for patients with hip and spine fractures, respectively. Regarding multiple fractures, 283 (16%), 64 (4%), and 25 (1%) had two, three, and four or more fractures, respectively.

**Table 1:** Demographic details of study population.

Patient Group	*n* Patients with at least One Fracture at Any Site	Mean Age at Start of Glitazone Exposure (y)^a^	*n* Fractures before Glitazone Exposure^b^	Duration (mean y) of Follow up Pre-Glitazone Exposure^a^	*n* Fractures during Glitazone Exposure^b^	Duration (Mean y) of Glitazone Exposure^a^
Any glitazone	1,819	62.0 (12.8)	1,546	9.5 (4.6)	720	2.3 (1.7)
Female	990	65.4 (12.1)	825	9.2 (4.6)	445	2.4 (1.7)
Male	829	57.9 (12.3)	722	9.8 (4.6)	274	2.3 (1.7)
Rosiglitazone only	1,356	62.2 (13.0)	1,138	9.4 (4.6)	542	2.3 (1.7)
Pioglitazone only	389	61.7 (12.3)	347	9.6 (4.5)	149	2.3 (1.6)

a Standard deviation given in parentheses.

b Some patients had more than one fracture.

Absolute rates of fracture are uninformative in this study since selection is restricted by design to patients who have had a fracture. The crude rate ratio for any fracture at any site, comparing thiazolidinedione exposed versus unexposed period was 1.70 (95% confidence interval [CI] 1.53–1.88), but this does not take into account the fact that each participant is older in the exposed period. We detected an annual increase in the rate of all fractures of approximately 5% when the effect of age was considered alone (rate ratio for a 1-y increase in age = 1.05, 95% CI 1.04–1.06). After adjustment for age in single year bands, the adjusted rate ratio for the association between thiazolidinediones and fracture was 1.43 (95% CI 1.25–1.62) comparing exposed with unexposed periods (Table 2). There was no evidence that the association varied by sex; the age-adjusted rate ratio for all fracture types amongst patients receiving any thiazolidinedione was 1.42 (1.20–1.69) for females and 1.44 (1.18–1.77) for males. When thiazolidinedione exposure was stratified by duration, the rate ratio for all fractures increased steadily from 1.26 (1.07–1.47) during the first year to 2.00 (1.48–2.70) during years four to seven. Fracture-site specific analyses for all thiazolidinedione users revealed a rate ratio of 1.28 (1.05–1.56) for arm, wrist, hand, or foot fractures, 2.09 (1.29–3.40) for hip fractures, and 2.72 (1.29–5.73) for spine fractures. There was no evidence that the rate ratio varied by gender. In patients who only received rosiglitazone, the rate ratio for fracture at any site was 1.49 (1.28–1.74), and amongst those who only received pioglitazone the rate ratio was 1.26 (0.95–1.68) (Table 3). A test for interaction showed no evidence that the effect varied by thiazolidinedione type (*p* = 0.47). The rate ratios for fractures at specific sites were also similar for each thiazolidinedione (Table 3).

**Table 2:** Case-series analysis for thiazolidinediones: association between exposure to any thiazolidinedione and fractures.

Fracture Site	Exposure	Patient Years	*n* Fractures	Age-Adjusted Rate Ratio for Fracture (95% CI
Fracture at any site				
All patients (*n* = 1,819)	Unexposed periods	17,221	1,546	Baseline
	Exposed periods (all)	4,240	720	1.43 (1.25–1.62)
Thiazolidinedione exposure period^a^	0–1 y (*n* = 1,819)	1,600	235	1.26 (1.07–1.47)
	1–2 y (*n* = 1,325)	1,087	179	1.49 (1.24–1.79)
	2–3 y (*n* = 874)	714	127	1.70 (1.37–2.12)
	3–4 y (*n* = 564)	446	104	2.31 (1.80–2.97)
	4–7 y (*n* = 342)	392	75	2.00 (1.48–2.70)
Females (*n* = 990)	Unexposed periods	9,103	824	Baseline
	Exposed periods	2,357	446	1.42 (1.20–1.69)
Males (*n* = 829)	Unexposed periods	8,118	722	Baseline
	Exposed periods	1,883	274	1.44 (1.18–1.77)
Foot, arm, wrist, or hand fracture				
All patients (*n* = 905)	Unexposed periods	8,599	735	Baseline
	Exposed periods	2,102	284	1.28 (1.05–1.56)
Females (*n* = 519)	Unexposed periods	4,837	411	Baseline
	Exposed periods	1,227	183	1.26 (0.98–1.62)
Males (*n* = 386)	Unexposed periods	3,762	324	Baseline
	Exposed periods	876	101	1.28 (0.93–1.77)
Hip fracture				
All patients (*n* = 150)	Unexposed periods	1,317	71	Baseline
	Exposed periods	394	87	2.09 (1.29–3.40)
Females (*n* = 105)	Unexposed periods	862	45	Baseline
	Exposed periods	280	66	2.09 (1.17–3.72)
Males (*n* = 45)	Unexposed periods	456	26	Baseline
	Exposed periods	113	21	1.90 (0.74–4.91)
Spine fracture				
All patients (*n* = 66)	Unexposed periods	624	41	Baseline
	Exposed periods	155	29	2.72 (1.29–5.73)
Females (*n* = 34)	Unexposed periods	311	21	Baseline
	Exposed periods	82	15	2.34 (0.77–7.13)
Males (*n* = 32)	Unexposed periods	313	20	Baseline
	Exposed periods	73	14	3.53 (1.18–10.58)

a Test for trend (*p*<0.01).

**Table 3:** Case-series analysis for thiazolidinediones: association between exposure to specific thiazolidinediones and fractures.

Fracture Site	Exposure	Patient Years	*n* Fractures	Age-Adjusted Rate Ratio for Fracture (95% CI)
Fracture at any site				
Rosiglitazone (*n* = 1,356)	Unexposed periods	12,772	1,139	Baseline
	Exposed periods	3,180	543	1.49 (1.28–1.74)^a^
Pioglitazone (*n* = 389)	Unexposed periods	3,747	347	Baseline
	Exposed periods	892	149	1.26 (0.95–1.68)^a^
Foot, arm, wrist, or hand fracture				
Rosiglitazone (*n* = 675)	Unexposed periods	6,446	539	Baseline
	Exposed periods	1,564	211	1.30 (1.03–1.64)
Pioglitazone (*n* = 188)	Unexposed periods	1,748	158	Baseline
	Exposed periods	446	61	1.43 (0.92–2.22)
Hip fracture				
Rosiglitazone (*n* = 115)	Unexposed periods	1,004	56	Baseline
	Exposed periods	310	66	1.80 (1.03–3.15)
Pioglitazone (*n* = 32)	Unexposed periods	282	13	Baseline
	Exposed periods	75	20	2.65 (0.81–8.70)
Spine fracture				
Rosiglitazone (*n* = 53)	Unexposed periods	492	33	Baseline
	Exposed periods	132	24	3.13 (1.35–7.21)
Pioglitazone (*n* = 11)	Unexposed periods	107	7	
	Exposed periods	18	4	*

Patients exposed to both thiazolidinediones excluded.

a Test for interaction (*p* = 0.47).

*: Insufficient outcomes to calculate rate ratio.

Bias can be introduced to a self-controlled case-series analysis if follow up is curtailed because of the event of interest in a substantial number of cases (e.g., due to a fatal outcome). For this reason a sensitivity analysis was performed for any fracture at any site, excluding patients who died during their observation period (*n* = 155). The rate ratio for thiazolidinedione exposed versus unexposed periods in this subgroup was 1.39 (1.21–1.59). Fracture-site specific analyses also yielded very similar results when patients who died were excluded.

A series of further sensitivity analyses were conducted: (1) censoring follow up in November 2006, prior to publication of the ADOPT study results highlighting fractures as a possible risk with rosiglitazone [5]; (2) restricting follow up to 2000 onwards, when thiazolidinediones were first available in the UK; and (3) restricting analysis to 2 y prior to and post first thiazolidinedione prescription. These three sensitivity analyses had no material effect on our results.

The self-controlled case-series analysis was repeated using the same study population, but measuring the association between all fracture types and exposure to sulphonylureas rather than thiazolidinediones (Table 4). 694 relevant patients were identified and the rate ratio for fracture at any site amongst all patients prescribed a sulphonylurea was 0.84 (0.66–1.08). There was no evidence that the association varied with sulphonylurea exposure duration up to 7 y.

**Table 4:** Case-series analysis for sulphonylureas: association between exposure to any sulphonylurea and fractures.

Fracture at Any Site	Exposure	Patient Years	*n* Fractures	Age-Adjusted Rate Ratio for Fracture (95% CI)
All patients (*n* = 694)	Unexposed periods	4,117	490	Baseline
	Exposed periods (all)	2,408	348	0.84 (0.66–1.08)
Sulphonylurea exposure period^a^	0–1 year (*n* = 694)	597	102	0.89 (0.69–1.16)
	1–2 y (*n* = 520)	460	61	0.77 (0.56–1.05)
	2–3 y (*n* = 405)	357	53	0.94 (0.67–1.31)
	3–4 y (*n* = 305)	267	43	1.09 (0.76–1.59)
	4–7 y (*n* = 243)	465	62	1.01 (0.71–1.43)

a Test for trend *p* = 0.50; sum of patient years and number of fractures in analysis stratified by exposure duration is less than in the main analysis as follow up censored at 7 y in stratified analysis.

## Discussion

In these data we have found an increased risk of fracture in users of thiazolidinediones. Our results suggest that the increased risk applies to both men and women and is observed at a wide range of fracture sites. In addition, we were able to look at the effects of treatment duration on fracture risk, and our results suggest the risk may increase steadily with exposure duration up to 7 y. The risk estimate with pioglitazone was comparable to that seen with rosiglitazone at each fracture site, although the results for pioglitazone failed to reach statistical significance given that relatively fewer patients registered in the GPRD receive pioglitazone. While a clear mechanism for a deleterious effect of thiazolidinediones on bone has not been identified, a recent review has highlighted a number of preclinical studies in which rosiglitazone was associated with an increase in bone loss [12].

A key advantage of this study was the use of the self-controlled case-series design, which is much less prone to problems of confounding than traditional case-control or cohort designs. Between-patient differences are of little relevance since the risk comparisons are made entirely within patients. Also, the self-controlled case series is often more efficient in terms of power than other observational study designs and so more precise estimates of effects can be made.

Other main strengths of the study were that it was large and statistically powerful. Our study was based on routine clinical data from the UK GRPD, which is representative of the population of the UK and so the results are likely to be highly generalisable. The validity of fracture recording in the GPRD has been shown to be high, and drug-related fractures have been the subject of several studies within this database [13]–[15]. A potential weakness might relate to the quality of the clinical data. Drug prescriptions in the GPRD are generated by practice computers, ensuring the accuracy of the electronic prescribing records. Prescription data were highly detailed and recorded prior to people developing fracture so there was no potential for recall bias. It is possible that some patients were not taking their prescribed thiazolidinediones during periods we assigned as exposed; however, we believe the likelihood of widespread low adherence in patients with a serious illness such as diabetes is unlikely and this would tend to dilute any true effect estimate for thiazolidinedione exposure.

We also acknowledge there are inherent difficulties in determining whether a recorded diagnosis in the GPRD is truly incident or a repeat record of a previous diagnosis. We aimed to do this by excluding from the analysis all fractures occurring within 6 mo of a previous fracture at the same site. This approach may lead to an overestimate in the number of incident fractures and so we conducted a sensitivity analysis where only the first ever fracture recorded for each patient was included. This made no material difference to our results.

The validity of the self-controlled case-series method relies on the independence of the decision to initiate treatment and the risk of the outcome. For this study, it requires that the decision to prescribe a thiazolidinedione is independent of whether a patient has ever had a fracture and their perceived risk of having a fracture at a later date. It also assumes the decision to start a thiazolidinedione is not temporally correlated with other exposures that influence the risk of fractures (e.g., corticosteroid therapy). If these assumptions are invalid, the results presented here could reflect confounding whereby a change in the underlying risk of fractures is temporally associated with a decision to change a patient's oral antidiabetic therapy. Although it seems implausible that the decision to prescribe a thiazolidinedione would be related to a change in the underlying risk of fracture, we conducted an analysis using exposure to sulphonylureas as a control to investigate this further. This analysis shows what happens to a patient's risk of fracture on commencing a new class of oral antidiabetic, and we found no evidence of an increase in age-adjusted fracture risk. This finding suggests confounding is unlikely to explain the results seen with thiazolidinediones, and that the effect we report here may be a direct effect of the drug. One would need to think of a form of time-varying confounding that applied to commencing one form of oral antidiabetic treatment (glitazones) but not to another (sulphonylureas) to be concerned that the thiazolidinedione results are due to unmeasured time-varying confounding. The static results over time for sulphonylureas also suggest the analysis has successfully adjusted for age, otherwise we would expect to see the relative risk increasing with sulphonylurea exposure period. In addition, the sensitivity analysis restricting each patient's observation period to the 2 y before and after first thiazolidinedione prescription attempted to remove some of the effect of age from the analysis and gave similar results to the main analysis, which again suggests age was adequately accounted for in these analyses.

We explored whether the choice of observation period could have introduced bias to the results with sensitivity analyses. Publication of the ADOPT trial in 2006 first highlighted fracture as a possible risk with rosiglitazone and could have led to a change in prescribing habits. An analysis restricted to observations before publication had no material effect on our results. Secondly, thiazolidinediones first became available in 2000; an analysis restricted to observations from 2000 onwards also had no material effect on the results, suggesting our choice of observation period was robust to these factors.

Consideration must be given to other possible sources of bias. Patients hospitalized following a fracture could continue thiazolidinedione therapy but prescriptions issued in hospital would not be recorded in the GPRD and such periods would therefore be missing from our analysis. The resulting underestimate of thiazolidinedione exposed time would lead to an overestimate of the risk during exposed periods. We therefore looked for evidence of apparent thiazolidinedione discontinuation postfracture. Less than 1% of patients in our study discontinue their thiazolidinedione within 30 d of a fracture, indicating this is unlikely to be a major source of bias. Similarly, bias can be introduced with this form of case-series analysis if a substantial proportion of the events of interest are fatal. The exact cause of death can be difficult to establish using GPRD records. Nonetheless we noted that death was recorded for 155 patients in this study. Of these, 88 had their final fracture before starting a thiazolidinedione and only 11 (0.5%) had a fracture during the 30 d prior to death. This result suggests fatal fractures were rare in this study and therefore unlikely to lead to substantial bias. The sensitivity analysis excluding patients who died confirms this as the results were very similar to the main analysis.

Because the risk of fractures changes substantially as people become older, it is important that our analysis adequately controlled for the effect of age. We therefore adjusted for age at the date of fracture, in single-year bands. The self-controlled case-series method facilitates fine adjustments for age at every stage of the analysis by stratifying follow-up time on a combination of drug exposure and age. Of vital importance, patient age at first thiazolidinedione prescription varied widely, and so although each individual patient is older during their exposed period, both exposed and unexposed states are fully represented in each age band. We are confident that the increased risk of fractures detected with thiazolidinediones is unlikely to be the effect of residual confounding by age because the distinct lack of an association with sulphonylurea exposure suggests our approach successfully accounted for age.

The overall increased risk seen in our study is slightly smaller than the near doubling of risk seen in clinical trials, although this may reflect differences in treatment duration. We have found that the relative risk of fracture appears to increase with treatment duration, and the median treatment duration in the ADOPT study was 4 y [5], compared with a mean duration of 2 y in our study. Of most importance, the risk we have identified appears to be nonspecific in terms of fracture site or gender, in contrast with the trials that reported a risk predominantly in women, affecting bones in the hand, arm, wrist, and foot [5],[6]. However, these differences may be due to a lack of power in the clinical trials to detect fractures at less frequently affected sites, or in men, who generally have a lower rate of fractures than women [16].

The recently published RECORD randomized clinical trial of rosiglitazone versus metformin or a sulphonylurea also reported an increased risk of fractures associated with rosiglitazone treatment (overall risk ratio: 1.57, 95% CI 1.26–1.97) [17]. Fractures were more common in women than men, and upper/lower limbs were the sites most commonly affected. Very few hip and spine fractures were reported, precluding firm conclusions about any effect of glitazones at these sites. Although the study was not designed or powered to study fractures, the results appear broadly consistent with those reported here.

The large number of patients included in our study allowed us to look in detail at the effect of treatment duration, and our results suggest the increased risk of fractures is present within a year of starting thiazolidinedione treatment, and the relative risk appears to increase with continued treatment. This finding is of particular relevance as the absolute risk of fractures with serious sequelae (e.g., hip and vertebrae) increases dramatically with age [18], and an additional increasing risk associated with thiazolidinediones is likely to be of concern in patients at high baseline risk of fractures. Of note, the age at first thiazolidinedione prescription was 10 y greater in our study than the age at first exposure in the ADOPT trial of rosiglitazone and so the absolute increase in fractures would be substantially higher amongst this more elderly “real-world” population than the absolute increase seen in clinical trials. To demonstrate how our findings would affect patients with different underlying fracture risks we have taken age- and sex-specific fracture rates from the GPRD calculated by van Staa et al. [16] and applied the relative rate for all fractures of 1.43 measured in the present study. In men aged 50–55 y, treatment with a thiazolidinedione could be expected to increase the rate of all fractures by around 28 per 10,000 patient years. By contrast, in women aged 80–85 y the rate of fractures could increase by 125 per 10,000 patient years.

A recent case-control study based on the GPRD also identified an increased risk of fractures associated with thiazolidinedione use [18]. However, this study relied on comparisons between people rather than within person. The reasons why someone might be prescribed or not prescribed thiazolidinediones are likely to be multiple and complex. In addition, it is likely that at least some of the differences between people exposed and not exposed could be related to the risk of future health outcomes, including fractures. Between-person comparisons are therefore potentially prone to selection bias and confounding due to factors that are unknown or difficult to measure and control for. While broadly similar, the risk estimates in the study by Meier et al. were somewhat higher than those observed in our own study, possibly suggesting an element of residual confounding in the case-control study. It is notable that in the case-control study, only 47 of the people with fractures and 119 people in the control comparison group had been exposed to a thiazolidinedione as the study focused on a narrow range of fracture types. This result compares to the 1,819 people with fractures who had been exposed to a thiazolidinedione included in our study.

We have demonstrated an increased risk of fractures associated with thiazolidinedione treatment. The increased risk was observed with both rosiglitazone and pioglitazone, in both men and women, and for a wide range of fracture sites including the hip. The risk increases with treatment duration. These findings should be taken into consideration in the wider debate surrounding the possible risks and benefits of treatment with thiazolidinediones.

## Abbreviations

CI: confidence interval
GPRD: General Practice Research Database
